# Prognostic Utility of Combining VI-RADS Scores and CYFRA 21-1 Levels in Bladder Cancer: A Retrospective Single-Center Study

**DOI:** 10.3390/curroncol32080415

**Published:** 2025-07-24

**Authors:** Shunsuke Ikuma, Jun Akatsuka, Godai Kaneko, Hayato Takeda, Yuki Endo, Go Kimura, Yukihiro Kondo

**Affiliations:** Department of Urology, Nippon Medical School, 1-1-5 Sendagi, Bunkyo-ku, Tokyo 113-8603, Japan; s00-001@nms.ac.jp (J.A.); g-kaneko@nms.ac.jp (G.K.); s8053@nms.ac.jp (H.T.); y-endo1@nms.ac.jp (Y.E.); gokimura@nms.ac.jp (G.K.); kondoy@nms.ac.jp (Y.K.)

**Keywords:** bladder cancer, cytokeratin fragment 19, magnetic resonance imaging, reporting and data system

## Abstract

The Vesical Imaging Reporting and Data System (VI-RADS) is used to detect muscle-invasive bladder cancer, with emerging prognostic implications. Integrating imaging parameters with molecular biomarkers may improve risk stratification in bladder cancer. This study evaluated whether combining VI-RADS scores with serum cytokeratin fragment 19 (CYFRA 21-1) levels—a clinically relevant biomarker for bladder cancer—could improve overall survival (OS) prediction. We retrospectively analyzed 134 patients who underwent transurethral resection of bladder tumors, magnetic resonance imaging, and postoperative serum CYFRA 21-1 measurements. In total, 15 cancer-specific deaths were observed during follow-up. Receiver operating characteristic curve analysis identified optimal prognostic cut-off values: VI-RADS score ≥ 4 and CYFRA 21-1 level ≥ 1.8 ng/mL. The 1-, 2-, and 3-year OS in patients with both high VI-RADS scores and CYFRA 21-1 levels were 42.9%, 16.7%, and 8.3%, respectively, significantly lower than those in other groups (*p* < 0.001, 0.002, and 0.003, respectively). Multivariate Cox proportional hazards analysis demonstrated that such patients had the poorest OS (hazard ratio: 7.51; *p* = 0.002). This suggests that combining VI-RADS and CYFRA 21-1 improves prognostic accuracy in bladder cancer, demonstrating potential clinical utility by informing individualized treatment strategies; however, limitations include the retrospective study design and absence of external validation.

## Simple Summary

No widely accepted biomarkers for bladder cancer prognosis exist. To our knowledge, no previous study has combined magnetic resonance imaging (MRI) data with a serum biomarker for this purpose. We combined Vesical Imaging Reporting and Data System (VI-RADS) scores with serum cytokeratin fragment 19 (CYFRA 21-1) levels for prognostic prediction. This retrospective study examined 134 patients with bladder cancer after transurethral resection. Patients with both high VI-RADS scores (≥4) and CYFRA 21-1 levels (≥1.8 ng/mL) had a 3-year overall survival of 8.3% and were strongly associated with poor prognosis (hazard ratio = 7.51, *p* = 0.002). In subgroup analyses, this group showed significantly worse outcomes in both treated (HR = 4.80, *p* = 0.003) and untreated patients (HR = 35.98, *p* < 0.001), emphasizing the importance of early identification and intervention in high-risk patients. This combined model could identify high-risk bladder cancer patients, supporting earlier and more personalized treatment decisions.

## 1. Introduction

Bladder cancer (BC) is among the top 10 most common cancer types worldwide, with approximately 550,000 new cases reported annually in 2020 [1]. At diagnosis, approximately 30% of patients with BC present with muscle-invasive BC (MIBC) and 5% with metastatic BC (MBC). The 5-year cancer-specific survival rate for MIBC patients is approximately 65%, and the prognosis is poor [2]. Therefore, accurate diagnosis is important for the effective treatment of BC.

The Vesical Imaging Reporting and Data System (VI-RADS) is widely used to predict MIBC and has shown high diagnostic accuracy [3]. However, its prognostic value remains under investigation and is mainly supported by studies involving post-cystectomy patients [4]. These limitations highlight the need for additional prognostic tools that can be applied prior to definitive treatment.

Serum tumor markers have gained increasing attention in recent years as minimally invasive tools for risk stratification in solid tumors, including BC. Their potential to reflect tumor biology, tumor burden, and treatment response makes them useful adjuncts to imaging-based assessments. A recent review emphasized the potential clinical utility of serum tumor markers in MIBC, highlighting their role in diagnosis, prognosis, and disease monitoring [5]. We specifically investigated cytokeratin fragment 19 (CYFRA 21-1) as a complementary biomarker to VI-RADS. Cytokeratins are proteins found in epithelial cells that indicate differentiation. As a soluble fragment of cytokeratin 19, CYFRA 21-1 is detectable in the serum and is elevated in various solid tumors, including BC [6,7,8]. In other cancer types, a combination of imaging diagnostics and serum biomarkers has been used to improve the accuracy of diagnosis and prognosis prediction [9]. However, to the best of our knowledge, no previous study has evaluated the prognostic utility of combining VI-RADS and CYFRA 21-1 in BC. Therefore, this study aimed to examine the prognostic utility of this multimodal approach in BC. We hypothesized that a combination of VI-RADS and serum CYFRA 21-1 levels would provide more accurate prognostic stratification than either marker alone.

## 2. Materials and Methods

### 2.1. Study Population

This retrospective, single-center study included patients with suspected BC who underwent preoperative magnetic resonance imaging (MRI), transurethral resection of bladder tumor (TURBT), and subsequent measurement of serum CYFRA 21-1 levels at Nippon Medical School Hospital between January 2019 and August 2023. Patients were identified through a review of institutional electronic medical records. One patient without pathological confirmation of urothelial carcinoma (UC) was excluded. The study was approved by the Institutional Review Board (IRB) of Nippon Medical School Hospital (Approval Number: F-2023-049) and conducted in accordance with the principles of the Declaration of Helsinki. The requirement for written informed consent was waived due to the retrospective nature of the study; however, all eligible patients were given the opportunity to opt out via the IRB website.

### 2.2. Image Acquisition

VI-RADS scoring was performed by two urologists—one with over 10 years of clinical experience and 5 bladder MRI interpretations, and the other with over 5 years of clinical experience and 3 bladder MRI interpretations. The overall VI-RADS score, ranging from 1 to 5, was assigned based on the combined interpretation of T2-weighted imaging (T2WI), diffusion-weighted imaging (DWI), and dynamic contrast-enhanced (DCE) MRI, following the recommendations described in the original VI-RADS proposal by Panebianco et al. [10]. In cases without DCE-MRI, the score was based on biparametric MRI using DWI and T2WI, as described by Noh et al. [11]. Disagreement between the two readers was resolved by consensus. In addition, a radiologist provided diagnostic examples to assist in the grading process. The two MRI readers were blinded to all outcome data, including pathological diagnosis and serum CYFRA 21-1 levels, to prevent interpretation bias. Additionally, there were no missing MRI data, pathological findings, or CYFRA 21-1 measurements in this study cohort.

### 2.3. Techniques and Procedures in Transurethral Resection of BC

TURBT was performed by resecting all visible tumor lesions along with two additional deep layers from the index area to ensure sufficient sampling. The specimens were fixed in 10% formalin and submitted for pathological evaluation. Muscle invasion was assessed by a uropathologist with over 20 years of experience.

### 2.4. Serum Collection

CYFRA 21-1 levels were measured using an electrochemiluminescence immunoassay (Elecsys CYFRA 21-1 Immunoassay; Roche Diagnostics GmbH, Mannheim, Germany). CYFRA 21-1 levels were measured after TURBT. This approach aimed to avoid confounding effects from the local tumor volume and better reflect systemic disease activity. Sera were collected postoperatively with a median of 1 day (mean, 10.2; range, 1–116 days), and 85.8% of patients had samples obtained within 3 days after TURBT.

### 2.5. Study Workflow and Statistical Methods

First, VI-RADS scores were evaluated by two urologists. We then constructed a receiver operating characteristic (ROC) curve based on VI-RADS scores and CYFRA 21-1 levels to predict 1-year survival. We divided the patients into four groups based on cut-off values: high and low VI-RADS scores and high and low CYFRA 21-1 levels. We summarized the clinicopathological characteristics of each group. Next, a Kaplan–Meier survival analysis was conducted to compare the overall survival (OS) among the four groups. To assess multicollinearity, collinearity diagnostics were performed using variance inflation factors (VIF) prior to multivariate Cox regression analysis. Finally, Cox proportional hazards regression was conducted to assess the prognostic impact of individual clinicopathological factors. Additional subgroup analyses were conducted to compare outcomes between treated and untreated patients, particularly within the highest-risk group versus all other groups. These analyses were also performed in patients without metastasis to evaluate the prognostic impact in localized disease. The treated group was defined as patients who received either systemic chemotherapy or radical cystectomy. A post hoc power analysis was performed based on the observed hazard ratio (HR), α = 0.05, and the proportion of high-risk patients.

### 2.6. Statistical Analyses

The diagnostic performance of the VI-RADS in the detection of MIBC was evaluated. The weighted kappa coefficient was used to assess inter-reader agreement. The cut-off value for CYFRA 21-1 levels was determined using Youden’s index. Cox regression analysis was performed to estimate the HR and 95% confidence interval (CI) of each variable. To avoid multicollinearity, the VI-RADS scores and CYFRA 21-1 levels were not included as continuous or separate covariates in the multivariate model. Instead, the combined risk group variable (based on both VI-RADS score and CYFRA 21-1 level stratification) was used as a single composite variable to represent prognostic classification. Statistical analyses were performed using SPSS version 29.0 (SPSS Inc., Chicago, IL, USA) and GraphPad Prism version 10.0 (GraphPad Software, San Diego, CA, USA) software (*p* < 0.05).

## 3. Results

**Figure 1:**
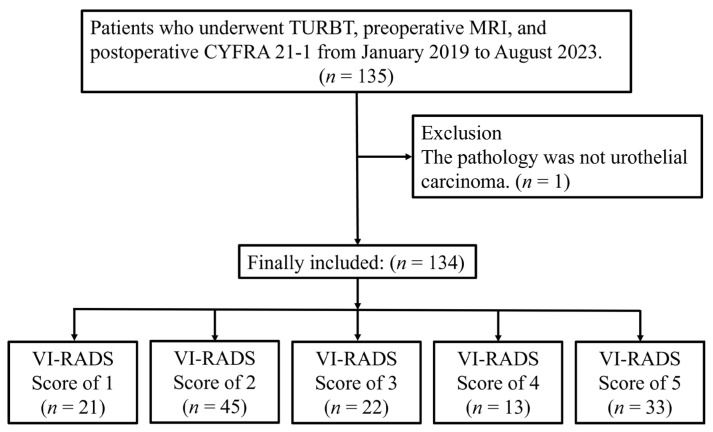
Patient selection flowchart for final analysis. TURBT, transurethral resection of bladder tumor; MRI, magnetic resonance imaging; CYFRA 21-1, cytokeratin 19 fragment 21-1; VI-RADS, Vesical Imaging Reporting and Data System.

A total of 135 patients who underwent TURBT, preoperative MRI, and measurement of serum CYFRA 21-1 between January 2019 and August 2023 were identified. One patient was excluded due to non-UC in the final pathology report. Consequently, 134 patients were included in the final analysis and categorized according to their VI-RADS scores, as shown in Figure 1.

In this study, the median follow-up duration was 20.0 months, during which 20 deaths (14.9%) were observed. A VI-RADS cut-off score of four showed an area under the curve (AUC) of 0.89, with 87.8% sensitivity, 89.2% specificity, 88.8% accuracy, 78.3% positive predictive value, and 94.3% negative predictive value. This VI-RADS score of four had better diagnostic performance than other VI-RADS scores. The agreement between the two readers was substantial, with kappa statistics of 0.84 (95% CI, 0.78–0.90) for the T2WI score, 0.88 (95% CI, 0.83–0.93) for the DWI score, 0.81 (95% CI, 0.73–0.89) for the DCE score, and 0.87 (95% CI, 0.81–0.93) for the VI-RADS score. Excellent agreement was observed between readers in the assessment of VI-RADS scores.

**Figure 2:**
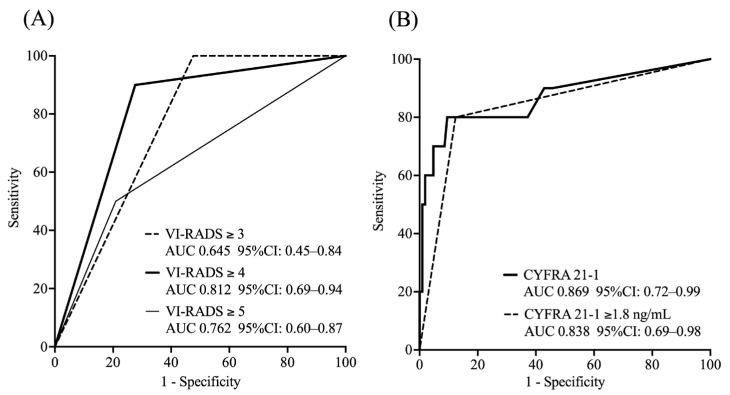
ROC curve analysis of VI-RADS score and CYFRA 21-1 levels for predicting 1-year overall survival. (**A**) VI-RADS score; (**B**) CYFRA 21-1 levels. VI-RADS, Vesical Imaging Reporting and Data System; AUC, area under the curve; CI, confidence interval; CYFRA 21-1, cytokeratin fragment 19.

ROC analysis showed that a VI-RADS score of four had a high predictive ability for 1-year survival (AUC: 0.812), whereas CYFRA 21-1 levels had a high predictive ability for 1-year OS (AUC: 0.869 as a continuous variable). Based on the ROC curve, the optimal cut-off value for CYFRA 21-1 was determined to be 1.8 ng/mL using the Youden index (value: 0.627), resulting in an AUC of 0.838 (Figure 2).

We classified the participants into four groups based on the combination of the VI-RADS score (≥4 or ≤3) and CYFRA 21-1 level (≥1.8 ng/mL or <1.8 ng/mL) as follows:

- Group 1: VI-RADS score ≥ 4 and CYFRA 21-1 level ≥ 1.8 ng/mL
- Group 2: VI-RADS score ≤ 3 and CYFRA 21-1 level ≥ 1.8 ng/mL
- Group 3: VI-RADS score ≥ 4 and CYFRA 21-1 level < 1.8 ng/mL
- Group 4: VI-RADS score ≤ 3 and CYFRA 21-1 level < 1.8 ng/mL

Table 1 shows the distribution of clinicopathological features according to each group. The median age of the patients was 73 years (interquartile range [IQR], 65–80 years). The blood tests showed mean CYFRA 21-1 levels of 2.01 ng/mL (IQR, 1.0–1.5; median 1.0 ng/mL). The histopathological findings showed that 93 (69.4%) patients had non-MIBC and 41 (30.6%) had MIBC. Seven (5.2%) patients had organ metastases, with some of them experiencing it in multiple organs. There were three lung metastases (2.2%), three liver metastases (2.2%), and three bone metastases (2.2%). Lymph node metastases were observed in eight patients (6.0%). Patients in Group 1 had average CYFRA 21-1 levels of 7.93 ng/mL and an average VI-RADS score of 4.75. Compared to the other groups, Group 1 showed the highest proportion of MIBC (93.8%), high-grade tumors (100%), organ metastasis (31.3%), and lymph node metastasis (37.5%). The 1-,2-, and 3-year OS rates in Group 1 were 42.9%, 16.7%, and 8.3%, respectively, indicating a markedly poor prognosis (*p* < 0.001, 0.002, and 0.003, respectively).

**Figure 3:**
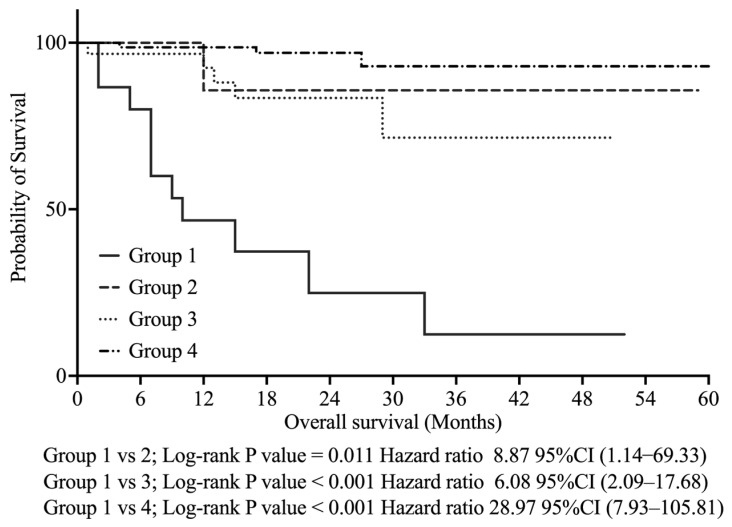
Kaplan–Meier curves for overall survival stratified by combined VI-RADS score and CYFRA 21-1 levels. Group 1: VI-RADS score ≥ 4 and CYFRA 21-1 level ≥ 1.8 ng/mL; Group 2: VI-RADS score ≤ 3 and CYFRA 21-1 level ≥ 1.8 ng/mL; Group 3: VI-RADS score ≥ 4 and CYFRA 21-1 level < 1.8 ng/mL; Group 4: VI-RADS score ≤ 3 and CYFRA 21-1 level < 1.8 ng/mL. VI-RADS, Vesical Imaging Reporting and Data System; CYFRA 21-1, cytokeratin fragment 21-1; CI, confidence interval.

As shown in Figure 3, Kaplan–Meier analysis revealed that Group 1 had significantly poorer OS than the other groups—Group 2 (*p* < 0.011, HR 8.87, 95% CI: 1.14–69.33), Group 3 (*p* < 0.001, HR 6.08, 95% CI: 2.09–17.68), and Group 4 (*p* < 0.001, HR 28.97, 95% CI: 7.93–105.81).

Univariate Cox regression analysis revealed that MIBC, high-grade histology, lymph node metastasis, organ metastasis, VI-RADS score ≥ 4, CYFRA 21-1 level ≥ 1.8 ng/mL, and their combination were significantly associated with a poor OS (Table 2). Multivariate Cox regression analysis showed that the combination of VI-RADS score ≥ 4 and CYFRA 21-1 levels ≥ 1.8 ng/mL was an independent predictor of a poor OS (HR 7.51, 95% CI: 2.12–26.62, *p* = 0.002), as shown in Table 2. The VIF values for VI-RADS ≥ 4, CYFRA 21-1 ≥ 1.8 ng/mL, and their combination (Group 1) were 2.44, 2.79, and 3.66, respectively, when all three were included in the model, indicating potential multicollinearity. After excluding the individual predictors and retaining only Group 1, all the VIF values dropped below 1.6, confirming acceptable levels. Consequently, VI-RADS ≥ 4 and CYFRA 21-1 ≥ 1.8 ng/mL were excluded from the final multivariate model and are indicated as “N/A” in Table 2. A post hoc power analysis, based on the observed hazard ratio (HR = 7.51), α = 0.05, and the proportion of high-risk patients (12%, defined as Group 1) indicated that at least 19 events were needed to achieve 80% power. Since 20 events occurred in our cohort, the study was considered adequately powered.

**Figure 4:**
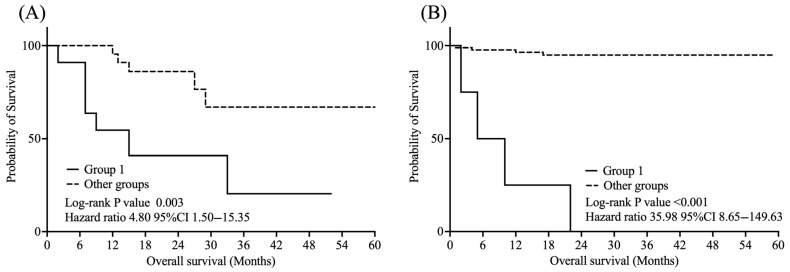
Kaplan–Meier curves for overall survival in subgroup analysis according to treatment status. Group 1: VI-RADS score ≥ 4 and CYFRA 21-1 level ≥ 1.8 ng/mL; (**A**) Treated subgroups. (**B**) Untreated subgroups.

As shown in Figure 4, among treated patients, Group 1 had significantly worse OS than the other groups (log-rank *p* = 0.003, HR = 4.80, 95% CI: 1.50–15.35). Similarly, in the untreated subgroup, Group 1 showed significantly poorer OS than the other groups (log-rank *p* < 0.001, HR = 35.98, 95% CI: 8.65–149.63).

**Figure 5:**
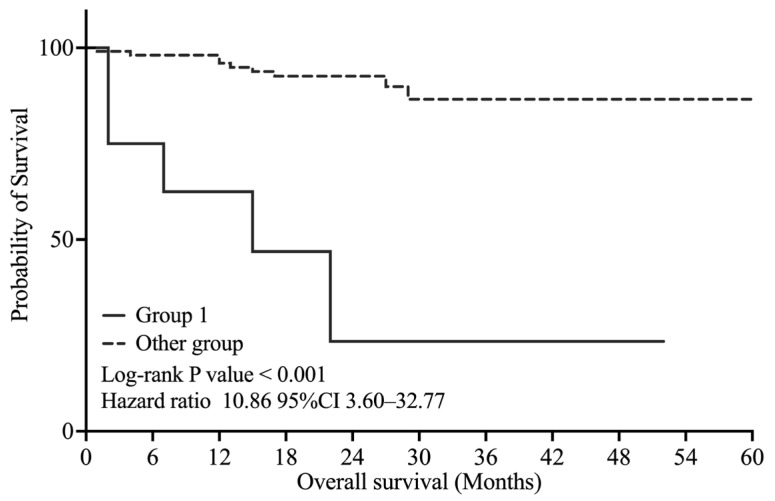
Kaplan–Meier curve for overall survival in the non-metastatic group. Group 1: VI-RADS score ≥ 4 and CYFRA 21-1 level ≥ 1.8 ng/mL.

Additionally, as shown in Figure 5, in the non-metastatic group, Group 1 also demonstrated significantly poorer OS than the other groups (log-rank *p* < 0.001, HR = 10.86, 95% CI: 3.60–32.77).

## 4. Discussion

This study assessed the clinical significance of VI-RADS scores and CYFRA 21-1 levels in predicting prognosis. Both high VI-RADS scores and elevated CYFRA 21-1 levels are significantly associated with poor prognosis. In this study, a combination of VI-RADS scores ≥ 4 and CYFRA 21-1 levels ≥ 1.8 ng/mL was strongly associated with poor prognosis in BC. To our knowledge, this is the first study demonstrating that the combination of VI-RADS and serum CYFRA 21-1 can effectively stratify BC patients by prognosis using a non-invasive, multimodal assessment. In particular, patients classified as high-risk based on both VI-RADS ≥ 4 and CYFRA 21-1 ≥ 1.8 ng/mL had a 3-year OS rate of only 8.3%, highlighting the model’s ability to identify this subgroup with extremely poor outcomes.

Previous studies have demonstrated the prognostic value of MRI for specific cases of BC [12]. The VI-RADS is a diagnostic tool specifically designed to predict whether BC has invaded the muscle layer via MRI, and it has been reported to be highly accurate [3]. In the present study, our VI-RADS scoring also showed high diagnostic performance for MIBC, with an AUC of 0.89. MIBC is essentially a poor prognostic factor, and its diagnosis may directly help in predicting poor outcomes in patients with BC. Recent studies have suggested that a higher VI-RADS score may be associated with worse oncological outcomes. Zhuang et al. [4] showed that patients with VI-RADS scores ≥3 had significantly worse OS (HR = 3.517, *p* = 0.003). Consistently, our study also showed that patients with VI-RADS scores ≥ 4 had significantly worse OS (HR = 9.38, *p* < 0.001). These findings suggest that high VI-RADS scores have potential utility not only in assessing muscle invasion but also in estimating the prognosis of patients with BC as an imaging biomarker. Previous studies have developed several prognostic nomograms to estimate the prognosis of BC. Shariat et al. [2] showed that clinicopathological features such as pathological T-stage, lymph node status, and tumor grade are associated with oncological outcomes. Based on these factors, they developed a prognostic model that has been widely cited and is primarily used after radical cystectomy. While these models have contributed to more refined risk stratification in BC, many still depend on the pathological information collected after radical cystectomy, limiting their applicability for perioperative decision-making around TURBT [13,14]. In contrast to conventional nomograms that rely on postoperative pathology, our model can be applied immediately after TURBT to support early risk stratification. Combining preoperative VI-RADS scores with serum CYFRA 21-1 levels enables the early identification of high-risk patients. This approach is particularly useful for selecting candidates for neoadjuvant chemotherapy or bladder-sparing strategies, where prompt decision-making is essential. Unlike models based solely on pathological staging, our method incorporates imaging and blood-based biomarkers, which may reflect tumor aggressiveness and occult metastasis. This imaging–biomarker model may complement existing post-cystectomy tools by offering personalized treatment strategies before definitive therapy.

In this study, CYFRA 21-1 levels also demonstrated significant value in estimating prognosis. Cytokeratin is overexpressed in BC [15]. Previous studies have demonstrated the accuracy of serum CYFRA 21-1 levels in diagnosing BC [16]. Daniel et al. [10] found increased CYFRA 21-1 levels in invasive UC. Andreadis et al. [17] reported that CYFRA 21-1 levels were significantly increased in patients with MBC. Moreover, an elevated serum CYFRA level was associated with poor prognosis. In our study model, the group with elevated CYFRA 21-1 levels after TURBT also had a lower 1-year OS, with an AUC of 0.87. The present study further attempted to combine CYFRA 21-1 levels with different VI-RADS score groups. Although CYFRA 21-1 level ≥ 1.8 ng/mL alone was a significant predictor of poor prognosis (HR: 9.13, *p* < 0.001), its combination with VI-RADS score ≥4 represented an even stronger prognostic factor (HR: 14.69, *p* < 0.001). Based on previous studies, we introduced CYFRA 21-1 as a complementary biomarker for advanced BC or MBC and developed a prognostic model combining this marker with VI-RADS. Although the possibility of lead-time bias cannot be completely ruled out, we intentionally measured CYFRA 21-1 levels after TURBT. We hypothesized that persistently elevated CYFRA 21-1 levels following local tumor resection may reflect the presence of residual local disease or undetected distant metastasis. This hypothesis is supported by prior reports demonstrating significantly elevated CYFRA 21-1 concentrations in MBC. For example, Andreadis et al. [17] reported elevated CYFRA 21-1 levels in 66% of metastatic cases, compared to only 7% of non-metastatic cases (*p* < 0.001). Similarly, in our cohort, patients with lymph node or visceral metastases had significantly higher mean CYFRA 21-1 levels (8.87 ng/mL) compared to those without metastasis (1.33 ng/mL, *p* < 0.001), suggesting that elevated post-TURBT CYFRA 21-1 levels may indicate the presence of advanced or metastatic disease. This supports the utility of CYFRA 21-1 as a complementary biomarker to VI-RADS and underscores the potential of the combined model to enhance risk stratification for poor prognosis in BC. To address the possibility that patients with metastatic disease may have biased the association between CYFRA 21-1 levels and prognosis, we performed a subgroup analysis restricted to non-metastatic cases. The results showed that the combination of VI-RADS and CYFRA 21-1 remained a strong prognostic indicator even in this non-metastatic group, supporting the robustness of the model across different disease stages. These findings suggest that using both biomarkers in combination may enable more accurate risk stratification than using either alone, potentially guiding decisions regarding earlier intervention or intensified follow-up in high-risk patients. Recent studies in other urologic malignancies have employed similar multimodal approaches integrating imaging and clinical factors including serum biomarkers. For instance, in clear cell renal cell carcinoma, a nomogram integrating contrast-enhanced CT radiomics features with clinical variables has been shown to improve survival prediction compared to clinical factors alone [18]. In prostate cancer, diagnostic models combining Prostate Imaging Reporting and Data System scores from multiparametric MRI with serum prostate-specific antigen levels have been shown to improve diagnostic accuracy compared to models using either modality alone [19,20]. These studies support the value of combining radiological and molecular data, reinforcing the rationale for our approach. Recent research has explored the integration of radiomics and clinical data for prognostic modeling in BC. For example, Haolin Huang et al. [21] developed a deep learning radiomics model based on MRI features combined with clinical variables, which achieved high accuracy in predicting 5-year recurrence risk in non-muscle-invasive BC. Similarly, another study employed CT-derived radiomics features combined with clinical data to develop a prognostic model using a gradient boosting machine algorithm, demonstrating significant predictive power for patient outcomes in MIBC [22]. The model demonstrated strong performance, supporting the utility of combining radiological and clinical variables for outcome prediction in BC. These findings provide further validation for the integration of imaging and clinical biomarkers in the BC setting. Building on these insights, our study may serve as a foundation for developing a more robust prognostic model that incorporates quantitative imaging features extracted via radiomics and serum CYFRA 21-1 levels. Such integration could enhance risk stratification and inform individualized treatment strategies in the future.

This study has several limitations. First, it was a retrospective, single-center analysis, which may limit the generalizability of the findings. Second, the proposed model has not been externally validated, and prospective studies are needed to confirm its utility in broader clinical settings. Third, while the MRI protocols followed standard VI-RADS guidelines, some variability in acquisition parameters may exist across patients, potentially affecting reproducibility. Another limitation is the potential variability in CYFRA 21-1 levels due to the timing of blood sampling, which was conducted postoperatively. Although most samples were obtained shortly after TURBT, the absence of preoperative measurements and the wide range in timing may have introduced measurement bias related to changes in tumor burden. Additionally, as the diagnostic stratification was based on imaging and biomarkers measured during the perioperative period of TURBT, the study may be subject to lead-time bias, whereby earlier detection or stratification could lead to an overestimation of survival duration. Moreover, as CYFRA 21-1 was not measured in all eligible patients—partly due to its lack of insurance coverage in Japan—some patients declined testing. Although this was not based on clinical factors, the possibility of selection bias cannot be completely excluded, although it is unlikely to have substantially influenced the results. Future studies with preoperative sampling and clearly defined diagnostic timepoints are warranted to minimize these sources of bias. Despite these limitations, the findings provide valuable insight into the potential of combining imaging and serum biomarkers for risk stratification in BC.

There are currently no widely established prognostic biomarkers specific to BC. The combination of imaging-based assessment using VI-RADS and the measurement of a serum biomarker such as CYFRA 21-1 provides a new multimodal approach. We propose this combination as a potential integrative biomarker bridging the radiological and molecular domains. The findings may provide a foundation for the development of next-generation prognostic tools for BC.

## 5. Conclusions

This study demonstrates that high VI-RADS scores and CYFRA 21-1 levels showed good predictive ability for a poor OS, suggesting their potential as prognostic markers in BC. Furthermore, the combination of these two factors effectively stratified patients into prognostically distinct subgroups. This multimodal approach, integrating imaging and serum biomarkers, may assist clinicians in early risk assessment and treatment decision-making. Future prospective studies with larger cohorts and longer follow-up are warranted to validate these findings and to explore their utility in treatment stratification and guiding individualized treatment strategies. In particular, multicenter prospective studies incorporating radiomics-based imaging features and serum biomarkers, such as CYFRA 21-1, may facilitate the development of a more robust and generalizable prognostic model.

## Figures and Tables

**Table 1:** Patient characteristics by VI-RADS and CYFRA 21-1-based classification.

	Total *n* = 134	Group 1 *n* = 16	Group 2 *n* = 8	Group 3 *n* = 30	Group 4 *n* = 80	*p* Value (Group 1 vs. Others)
Baseline Characteristics						
Mean age, (year)	73.0	74.6	77.9	72.3	70.9	0.286
IQR, (year)	65.0–80.0	67.2–82.5	72.3–82.5	65.8–79.3	63.0–79.8	
Gender						0.926
Male, (*n*)	110	13	6	24	67	
Female, (*n*)	24	3	2	6	13	
VI-RADS						
Score 1, (*n*)	21	0	2	0	19	
Score 2, (*n*)	45	0	4	0	41	
Score 3, (*n*)	22	0	2	0	20	
Score 4, (*n*)	13	4	0	9	0	
Score 5, (*n*)	33	12	0	21	0	
CYFRA 21-1						<0.001
Mean (ng/mL)	2.01	7.93	2.23	1.18	1.11	
IQR (ng/mL)	1.0–1.5	2.53–12.75	1.93–2.58	1.0–1.325	1.0–1.2	
Pathological Findings						
T-stage						<0.001
NMIBC, (*n*)	93	1	8	9	75	
MIBC, (*n*)	41	15	0	21	5	
Tumor grade						0.002
High, (*n*)	87	16	6	24	41	
Low, (*n*)	47	0	2	6	39	
CIS						0.165
Presence, (*n*)	13	0	2	3	8	
Absence, (*n*)	121	16	6	27	72	
Necrosis						0.052
Presence, (*n*)	26	6	1	10	9	
Absence, (*n*)	108	10	7	20	71	
Imaging Findings Metastasis						
Organ, (*n*)	7	5	0	1	1	<0.001
Lymph node, (*n*)	8	6	0	2	0	
Treatment Variables						
Systemic chemotherapy						<0.001
GC, (*n*)	29	9	0	15	5	
GCb, (*n*)	5	4	0	1	0	
Pembrolizumab, (*n*)	8	3	0	5	0	
Avelmab, (*n*)	5	1	0	3	1	
Enfortumab Vedotin, (*n*)	3	1	0	2	0	
Cystectomy, (*n*)	18	5	0	11	2	0.026

IQR, interquartile range; VI-RADS, Vesical Imaging Reporting and Data System; CYFRA 21-1, cytokeratin 19 fragment 21-1; NMIBC, non–muscle-invasive bladder cancer; MIBC, muscle-invasive bladder cancer; CIS, carcinoma in situ; GC, gemcitabine and cisplatin; GCb, gemcitabine and carboplatin.

**Table 2:** Cox regression analysis of prognostic factors for overall survival.

	Univariate HR (95%CI)	*p*-Value	Multivariate HR (95%CI)	*p*-Value
Age	1.02 (0.97–1.07)	0.461	1.00 (0.95–1.07)	0.893
MIBC	6.64 (2.54–6.64)	<0.001	1.35 (0.35–5.20)	0.659
High grade	10.95 (1.47–81.86)	0.020	4.62 (0.53–40.07)	0.165
Lymph node metastasis	5.43 (1.95–15.13)	0.001	0.73 (0.12–4.35)	0.733
Organ metastasis	7.77 (2.80–21.58)	<0.001	1.91 (0.40–9.19)	0.419
VI-RADS ≥ 4	9.38 (3.13–28.13)	<0.001	N/A	N/A
CYFRA 21-1 ≥ 1.8 ng/mL	9.13 (3.71–22.50)	<0.001	N/A	N/A
VI-RADS ≥ 4 + CYFRA 21-1 ≥ 1.8 ng/mL	14.69 (5.99–36.00)	<0.001	7.51 (2.12–26.62)	0.002

N/A: Not included in multivariate analysis due to multicollinearity with the combined variable. CYFRA 21-1, cytokeratin 19 fragment 21-1; MIBC, muscle-invasive bladder cancer; VI-RADS, Vesical Imaging Reporting and Data System.

## Data Availability

The data supporting the findings of this study are available from the corresponding author upon reasonable request. The data are not publicly available due to patient privacy restrictions.

## Abbreviations

The following abbreviations are used in this manuscript:

BC	Bladder cancer
MIBC	Muscle-invasive bladder cancer
MBC	Metastatic bladder cancer
VI-RADS	The Vesical Imaging Reporting and Data System
CYFRA 21-1	Cytokeratin fragment 19
MRI	Magnetic resonance imaging
TURBT	Transurethral resection of bladder tumor
UC	Urothelial carcinoma
IRB	Institutional Review Board
T2WI	T2-weighted imaging
DWI	Diffusion-weighted imaging
DCE	Dynamic contrast-enhanced
ROC	Receiver operating characteristic
HR	Hazard ratio
CI	Confidence interval
AUC	Area under the curve
IQR	Interquartile range
OS	Overall survival
VIF	Variance inflation factors
